# Structural and Morphology of ZnO Nanorods Synthesized Using ZnO Seeded Growth Hydrothermal Method and Its Properties as UV Sensing

**DOI:** 10.1371/journal.pone.0050405

**Published:** 2012-11-26

**Authors:** Nur Syafinaz Ridhuan, Khairunisak Abdul Razak, Zainovia Lockman, Azlan Abdul Aziz

**Affiliations:** 1 School of Materials and Mineral Resources Engineering, Universiti Sains Malaysia, Nibong Tebal, Penang, Malaysia; 2 NanoBiotechnology Research and Innovation, Institute for Research in Molecular Medicine, Universiti Sains Malaysia, Georgetown, Penang, Malaysia; 3 School of Physics, Universiti Sains Malaysia, Georgetown, Penang, Malaysia; Harbin Institute of Technology, China

## Abstract

In this study, zinc oxide (ZnO) nanorod arrays were synthesized using a simple hydrothermal reaction on ZnO seeds/n-silicon substrate. Several parameters were studied, including the heat-treatment temperature to produce ZnO seeds, zinc nitrate concentration, pH of hydrothermal reaction solution, and hydrothermal reaction time. The optimum heat-treatment temperature to produce uniform nanosized ZnO seeds was 400°C. The nanorod dimensions depended on the hydrothermal reaction parameters. The optimum hydrothermal reaction parameters to produce blunt tip-like nanorods (770 nm long and 80 nm in top diameter) were 0.1 M zinc nitrate, pH 7, and 4 h of growth duration. Phase analysis studies showed that all ZnO nanorods exhibited a strong (002) peak. Thus, the ZnO nanorods grew in a c-axis preferred orientation. A strong ultraviolet (UV) emission peak was observed for ZnO nanorods grown under optimized parameters with a low, deep-level emission peak, which indicated high optical property and crystallinity of the nanorods. The produced ZnO nanorods were also tested for their UV-sensing properties. All samples responded to UV light but with different sensing characteristics. Such different responses could be attributed to the high surface-to-volume ratio of the nanorods that correlated with the final ZnO nanorods morphology formed at different synthesis parameters. The sample grown using optimum synthesis parameters showed the highest responsivity of 0.024 A/W for UV light at 375 nm under a 3 V bias.

## Introduction

Zinc oxide (ZnO) nanostructures are receiving considerable interest because of their excellent electronic, optical, and photonic properties, as well as a wide bandgap of 3.2 eV that benefits short-wavelength optoelectronic applications. One-dimensional ZnO nanostructures (nanorods, nanowires, and nanotubes) can facilitate more efficient carrier transport because of decreased grain boundaries, surface defects, disorders, and discontinuous interfaces [1], [2]. Various methods of growing ZnO nanorods include vapor-liquid-solid deposition, pulsed laser deposition, and chemical vapor deposition [3]. However, the simplest, most economical, most energy-efficient method for synthesizing ZnO nanorods is by using the hydrothermal method [3]–[4]. Seeded substrates are used in the hydrothermal technique to achieve well-controlled morphology and growth direction of ZnO nanorods. Heterogeneous nucleation favors the growth of ZnO nanorods because the interfacial energy between crystals and substrate is usually smaller than the interfacial energy between crystals and solution [5]. Therefore, pre-coating the substrate with seed materials similar to nanocrystals can effectively control nanorods growth and morphology. Several works have used seeded substrates to produce ZnO nanorods.

Several methods have been employed to produce seeds such as thermal evaporation [2]–[3], metal organic chemical vapour deposition (MOCVD) [6]–[8], spin coating [9], and sputtering [10]–[14]. Li et al. [10] and Song et al. [11] deposited ZnO on silicon (Si)- substrate by using RF sputtering followed by hydrothermal reaction without any heat treatment, and they obtained columnar ZnO nanorods. These results show the importance of the heat treatment of ZnO seed layers in forming ZnO nanorods using the hydrothermal method. In our previous work, ZnO nanorods have been grown on a seeded Zn sheet, and the optimum ZnO seed formation was obtained after oxidation at 300°C [3]. Above 300°C, ZnO flakes instead of spherical ZnO seeds form on the Zn sheet. For a Zn thin film sputtered on PTFE substrate, thermal oxidation was performed at 300°C followed by hydrothermal reaction to grow ZnO nanorods. In both works, the potential applications of ZnO nanorods have not been investigated because of substrate unsuitability. Liu et al. [14] used ZnO deposited on Si substrate by ion beam sputtering followed by annealing at 200–600°C. The ZnO nanorod length increased with increased temperature, reaching 501 nm for seeds annealed at 600°C. This high annealing temperature can limit future applications of ZnO nanorod because the ZnO buffer layer degrades. This degradation can be induced by interdiffusion between the buffer layer and Si substrate, and also by the thinning of the buffer layer because of evaporation at high temperature [15]. Although Liu et al. [14] obtained almost similar results on the effect of the heat treatment of a seeded substrate; the effect of hydrothermal reaction parameters has not been studied. The electrical properties of the produced ZnO have also not been reported.

Ultraviolet (UV) sensing is widely used in various applications, including fire alarms, UV source monitoring, space technology, and environmental monitoring [16]–[17]. ZnO nanorods have been used as a base for UV photodetectors [14], [17]–[19]. ZnO nanorods can enhance UV-sensing properties because of their large surface area and superior electron transport properties [17]. In previous works [13], [16]–[20], high applied voltage biases (5–10 V) were used to obtain a photocurrent in the order of 10^−4^ A compared with the present study, which only used 3 V to obtain same order of current. Mamat et al. [17] measured a low photocurrent value of 4.68×10^−4^ A at a forward voltage of 10 V compared with our work (9.73×10^−4^ A at a forward voltage of 3 V).

The present study reports the fabrication and characterization of ZnO nanorods by a hydrothermal method using seeded ZnO substrates. The effects of hydrothermal parameters (including heat-treatment temperature, zinc nitrate (Zn(NO_3_)_2_) concentration, pH of hydrothermal bath solution, and hydrothermal reaction time) on ZnO nanorod formation were systematically studied. The UV-sensing properties of all samples were correlated with their structure and morphology. The final morphology and crystal structure of ZnO nanorods were found to affect the UV-sensing properties. Although several works have reported improvements in UV-sensing properties when using ZnO nanorods [13], [17]–[19], to the authors’ knowledge, reports on using ZnO nanorods produced at different hydrothermal parameters are very limited. Therefore, the main objective of this work is to determine the optimum parameters to grow ZnO nanorods via hydrothermal method using seeded substrates that consequently provide the best UV sensing properties.

### Experimental Details

Si substrates were cleaned by a standard cleaning method (Radio Corporation of America). Approximately 200 nm ZnO thin film was deposited on n-type Si (100) using RF magnetron sputtering at room temperature. The samples were then heat treated in air at various temperatures (250–450°C) for 10 min to obtain ZnO seeds for hydrothermal growth. The ZnO seeded samples were placed in screw-capped bottles containing 1∶1 molar ratio of Zn(NO_3_)_2_, and hexamethylamine (HMT) as a precursor solution. The hydrothermal reaction was performed in a preheated oven at 80°C for 4 h. Several synthesis parameters were studied to observe the morphological changes of the ZnO nanorods. These parameters were Zn(NO_3_)_2_ concentration (0.05–0.4 M), pH (3–13), and hydrothermal reaction time (1–24 h). The hydrothermal bath pH was adjusted with 1 M HCl or 1 M NaOH. The synthesis parameters are listed in Table 1. After hydrothermal reaction, the samples were cleaned with MilliQ water to eliminate residual salts and dried in air. An X-ray diffractometer (Bruker AXS D8 Advance XRD) with Cu_Kα_ radiation was used to determine the phases in the samples. The morphology of the samples was observed using a field-emission scanning electron microscopy (FESEM) system.

**Table 1:** Synthesis parameters of ZnO nanorods using hydrothermal method.

	Seed-heat-treatment temperatures	Zn(NO_3_)_2_ concentration	Hydrothermal bath solution pΗ	Hydrothermal growth duration
Seed-heat-treatment temperatures	250–450°C	+	+	+
Zn(NO_3_)_2_ concentration	+	0.05–0.4 M	+	+
Hydrothermal bath solution pΗ	+	+	pH 3–pH 13	+
Hydrothermal growth duration	+	+	+	4–24 h

+ is denoted as constant parameters.

**Figure 1:**
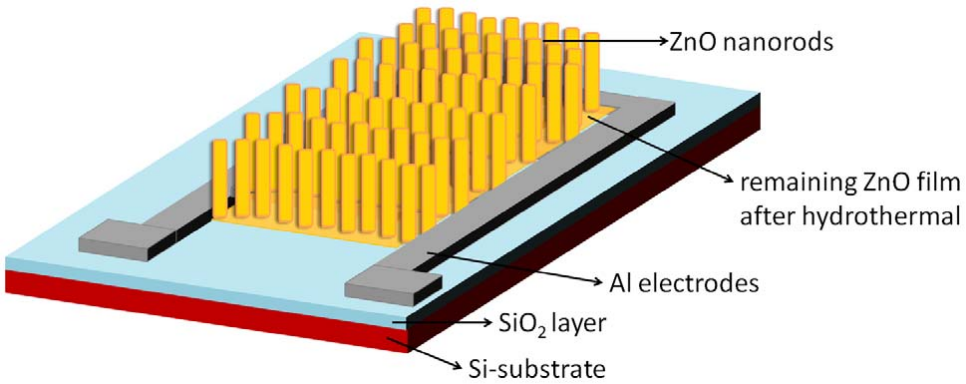
Schematic illustration of a UV photodetector with ZnO nanorod arrays. The UV source was placed 5 cm above the device.

A 150 nm-thick aluminum (Al) film was deposited onto the cleaned Si-substrate by thermal evaporation. Standard lithography and etching were then performed to obtain the interdigitated contact pattern for ohmic contact. The fingers of Al contact electrodes were 3 mm long and 0.35 mm wide, with 0.15 mm spacing between each finger. The effective surface area of the entire device used was 5.5 mm × 5.5 mm. A schematic drawing of the fabricated UV sensing device with ZnO nanorods is shown in Figure 1.

A light-emitting diode with a wavelength (*λ*) of 375 nm was used as a UV illumination source with a 40 mW optical power (*P*_op_). The UV source was placed 5 cm above the samples. The room-temperature current–voltage (*I*–*V*) characteristics of the devices were then measured using Keithley 237 source measure unit (SMU). The experiment was carried out under atmospheric pressure in darkness and UV illumination conditions.

**Figure 2:**
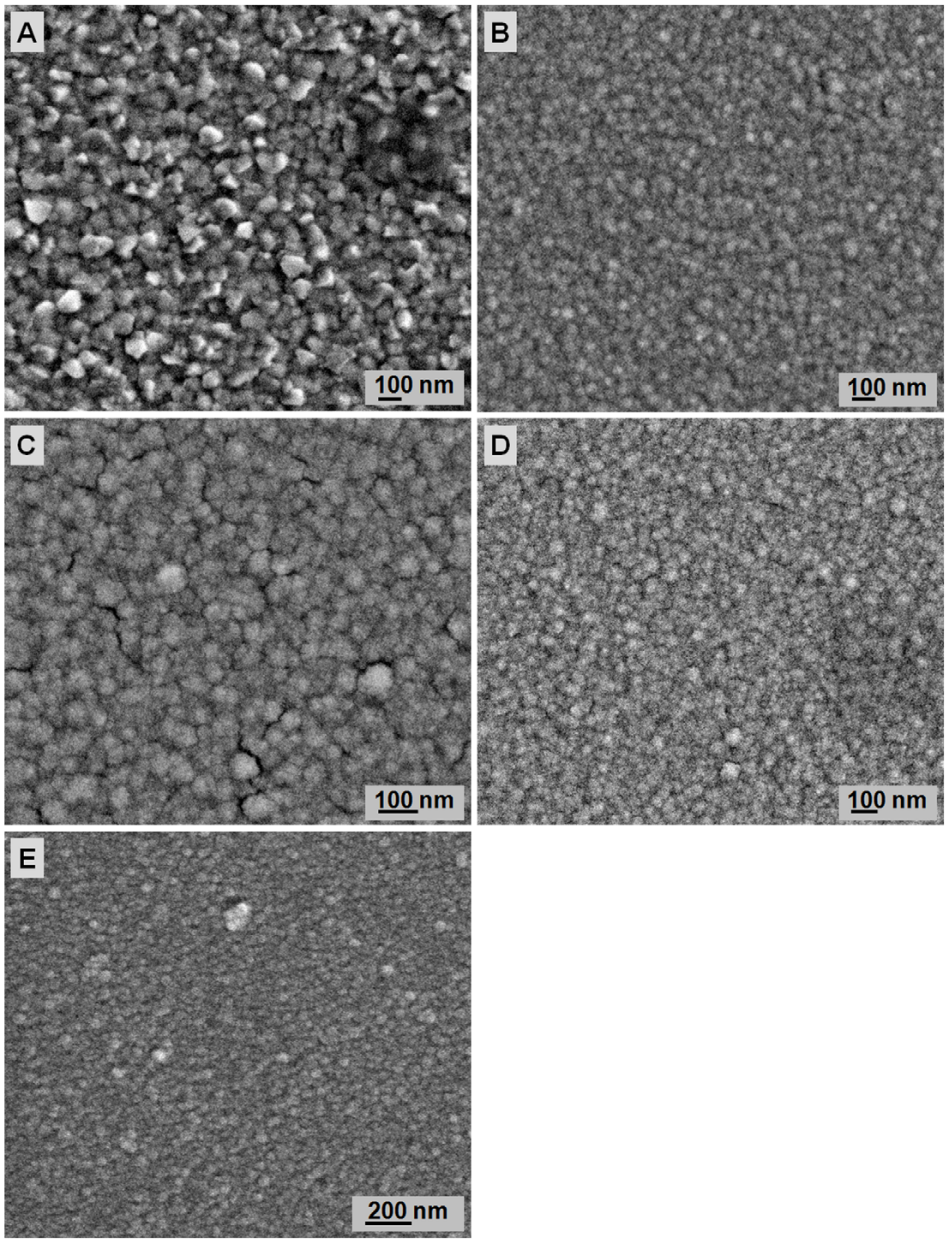
Surface morphologies of ZnO seeds heat treated at different temperatures: A) 250, B) 300, C) 350, D) 400, and E) 450°C.

## Results and Discussion

### 3.1 Effects of Different Seed-heat-treatment Temperatures

The surface morphology of ZnO films deposited on Si substrates after different heat-treatment temperatures is shown in Figure 2. After heat treatment at 250°C, the surface of ZnO films is covered with dispersed particulate grain size and an average diameter of ∼90 nm. The surface morphology of ZnO seed layer transforms from a flaky structure at 250°C to a circular structure with increased heat-treatment temperature from 300°C to 450°C. The ZnO seed size decreases with increased heat-treatment temperature. At high-temperature heat treatment, the seed layer particles are more mobile; they tend to dislocate and pile up to reduce the strain energy of the system. The rearrangements of these dislocations into low-angle-tilt boundaries lead to the formation of subgrains. At high temperatures, atoms vibrate at their lattice positions and exchange energy with neighboring atoms. As the atoms attain sufficient energy, they diffuse to achieve the lowest strain energy and form small seeds. Thus, smaller grains form with increased heat-treatment temperature. To achieve excellent nanorod array formation by the hydrothermal method, ZnO seeds must have uniform size and distribution. The ZnO seeds must not be too compact and be able to provide spaces for ZnO nanorods growth during a hydrothermal reaction. Based on the FESEM images in Figure 2, the ZnO seeds heat treated at 400°C shows the best seeds for ZnO nanorods formation by hydrothermal reaction.

**Figure 3:**
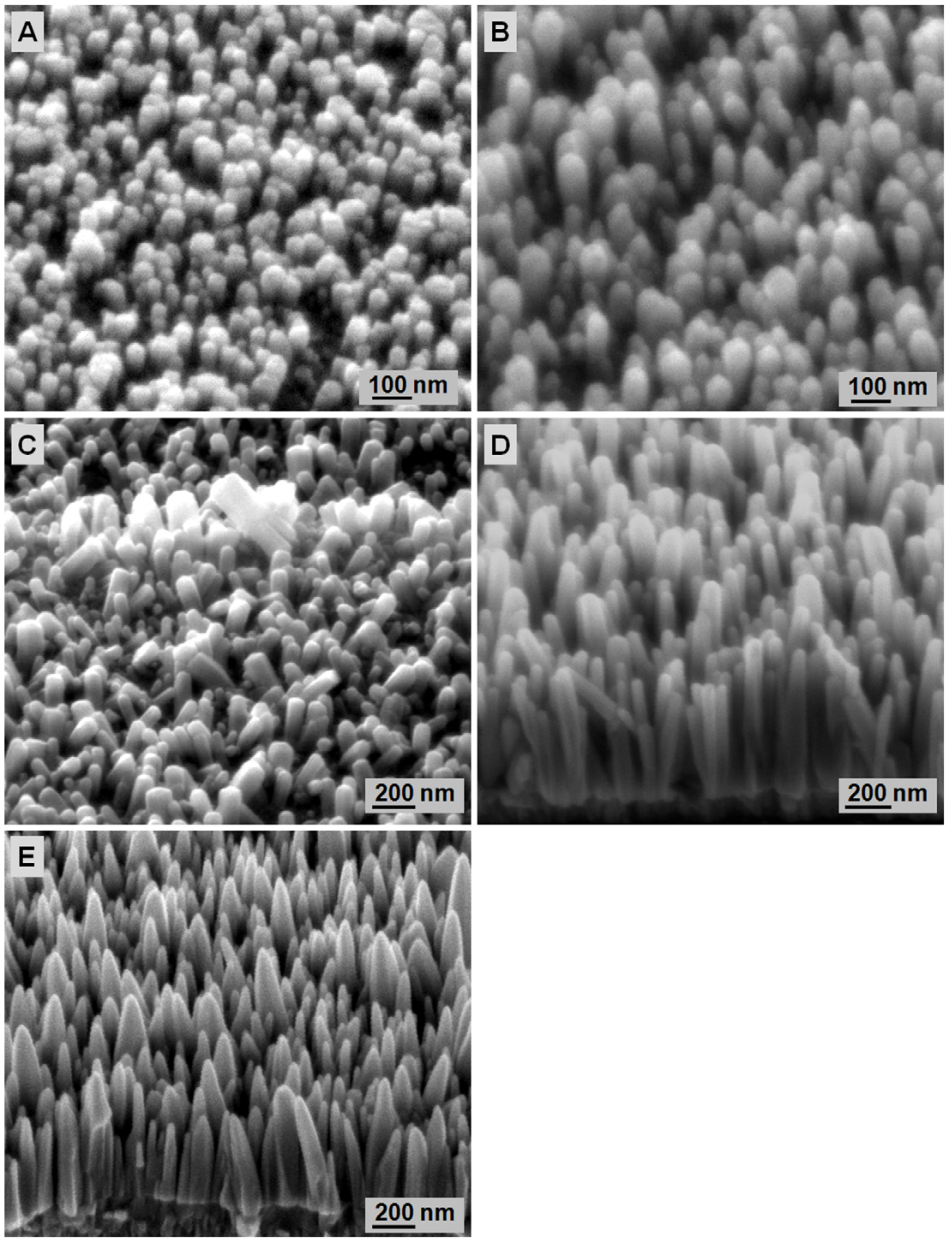
FESEM images of ZnO nanorods grown on seeds heat treated at different temperatures: A) 250, B) 300, C) 350, D) 400, and E) 450°C. The samples were hydrothermally grown for 4 h at 80°C.

The heat-treated seeded substrates were then subjected to hydrothermal growth at 80°C for 4 h to investigate the effect of ZnO seeds on ZnO nanorods formation. Figures 3A–3E show ZnO nanorods growth on ZnO seed substrates heat treated at different temperatures. The average lengths of ZnO nanorods are 105, 184, 231, 742, and 605 nm for ZnO seeds heat treated at 250, 300, 350, 400, and 450°C, respectively. The average diameter of the ZnO nanorods gradually increases from 64 nm to 80 nm with increased heat-treatment temperature of the seed layer. The increase in ZnO nanorods length is due to increasing reactivity of the seed layer surface during the nucleation stage [14]. The ZnO crystal structure consists of a number of alternating planes composed of coordinated Zn and O atoms along the c-axis direction. Generally, the Zn-terminated [0001] is at the top surface of the ZnO crystal structure, whereas the O-terminated [000)] is at the bottom surface, causing a normal dipole moment and a polar surface of ZnO seed layer [14]. The presence of two charges (Zn^2+^ and OH^−^ ions) in the hydrothermal solution results in attraction toward the polar surface of the ZnO seed layer. Consequently, Zn(OH)_2_ forms and produces ZnO upon dehydration. The increased heat-treatment temperature can improve the reactivity of the seed layer surface. This reactivity is stronger than the electrostatic interaction of the polar surface of the ZnO seed layer with the charges of Zn^2+^ and OH^−^ because of the energy supplied during heat treatment. Therefore, more Zn^2+^ and OH^−^ ions are diffused on the ZnO seeds, which results in increased ZnO nanorods length with increased heat-treatment temperature of the seed layer from 250°C to 400°C. However, further increase annealing temperature of the seed layer to 450°C results in decreased ZnO nanorods length at 450°C. Therefore, a threshold of annealing temperature for the ZnO seed layer that can be due to structural deterioration must exist.

**Figure 4:**
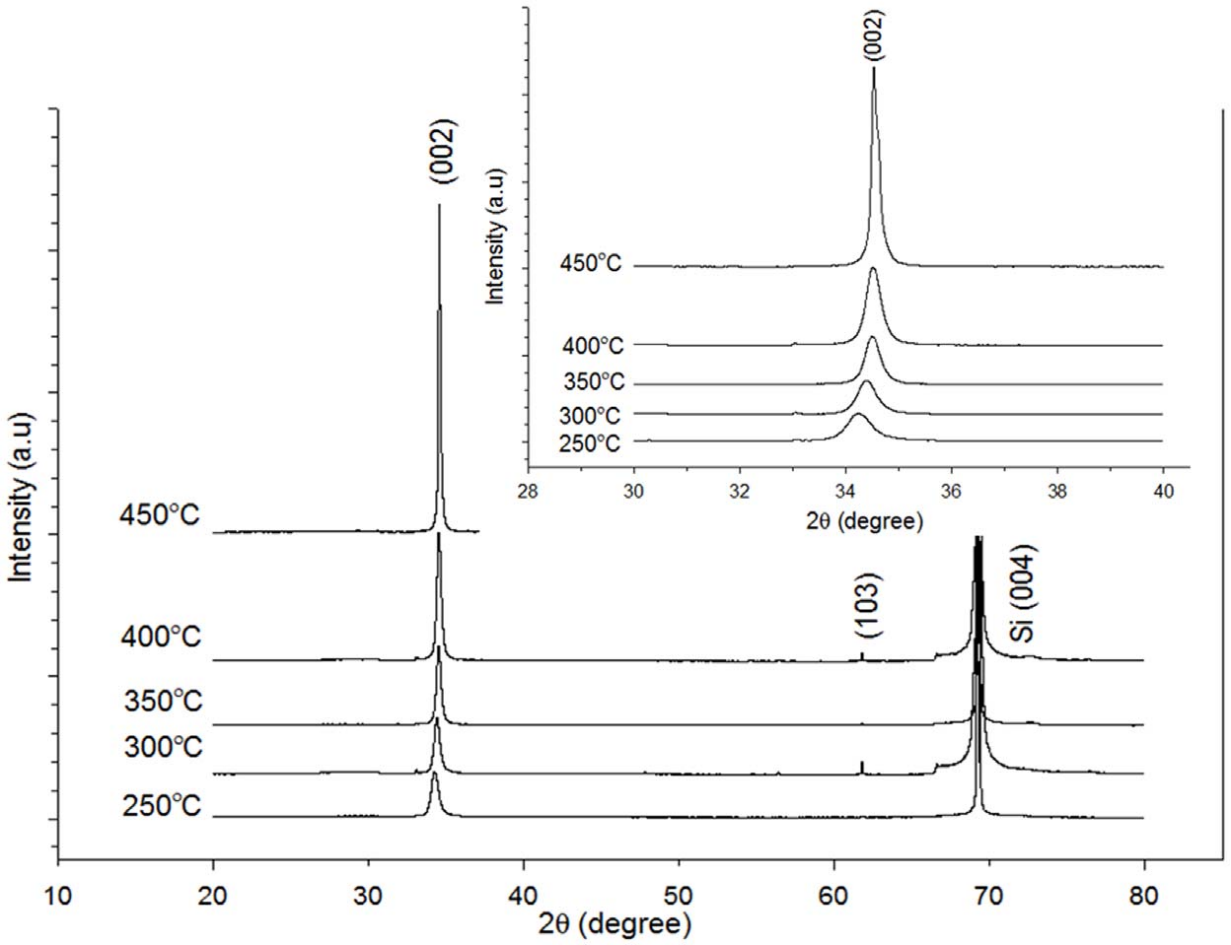
X-ray diffraction patterns of ZnO nanorods formed on seeds heat treated at different temperatures: A) 250, B) 300, C) 350, D) 400, and E) 450°C. The samples were hydrothermally grown for 4 h at 80°C. The insets show the (0 0 2) diffraction of ZnO formation at 2θ = 30°–40°.

**Figure 5:**
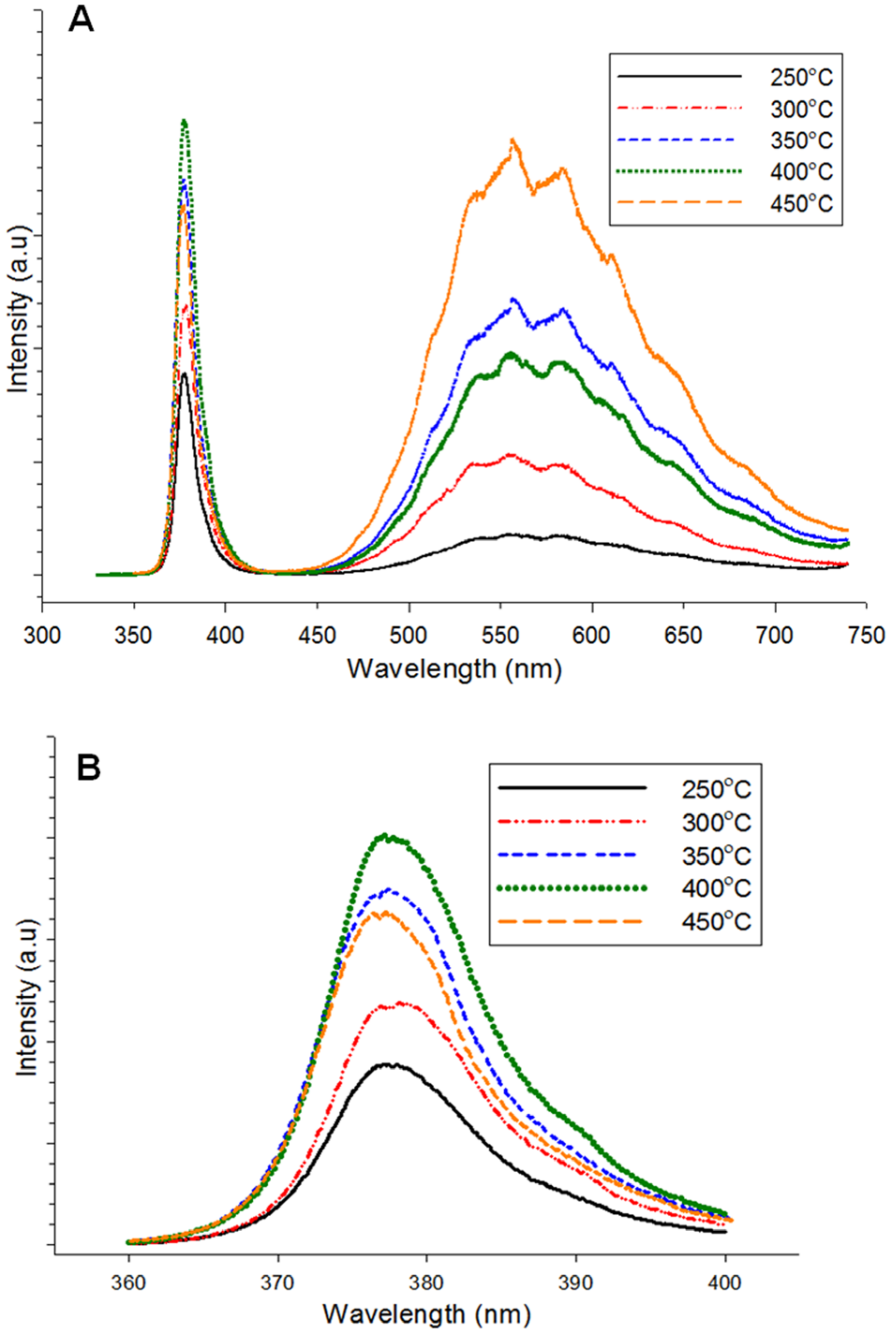
Room-temperature photoluminescence spectra of ZnO nanorods formed on seeds heat treated at different temperatures.

Figure 4 shows the X-ray diffraction (XRD) spectra of ZnO nanorods with varying heat-treatment temperatures of ZnO seeds. Two peaks corresponding to wurtzite ZnO with a strong (002) peak and a small (103) peak at 2*θ* = 34.4° and 61.7°, respectively, are observed. The (002) peak intensity increases with increased annealing temperature. When the annealing temperature increases, more energy is supplied, thereby causing Zn and O atoms to move into the proper sites. Consequently, the crystal quality is improved to reduce the Gibbs free energy and high-quality ZnO crystals are formed following thermal equilibrium [15]. However, the heat-treatment temperature of the seed layer only affects the crystal quality of the seed layer before hydrothermal reaction. The crystal quality of ZnO nanorods formed after hydrothermal reaction is not affected. Figure 2 shows that increased heat-treatment temperature produces smaller ZnO seeds. A larger ratio of the surface area to volume increases the surface area for the reaction to take place. Thus, the ZnO growth rate increases. The inset in Figure 4 shows the (002) diffraction of ZnO nanorods at 2*θ* = 30°–40°. The peak shifts to a slightly higher 2*θ* angle from 34.2° to 34.6° at a higher temperature of heat-treated seeds. The result may be due to the release of intrinsic strain induced by the presence of residual strain resulting from imperfections within the crystalline lattice, including vacancies, stacking faults, and interstitials. According to Shin et al. [7], the lattice mismatch between ZnO film and Si-substrate causes structural defects. Thus, annealing the ZnO film deposited on Si-substrate removes the intrinsic strain because the Zn and O atoms are perfectly stacked in order.

**Figure 6:**
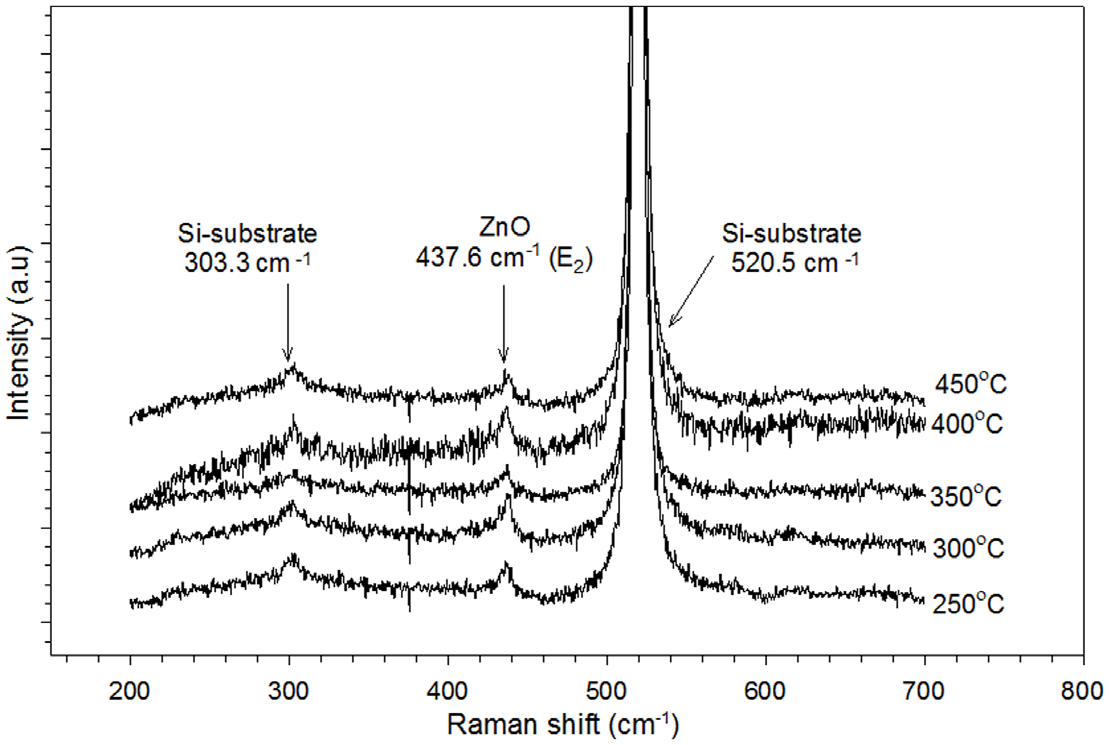
Raman spectra of grown ZnO nanorods formed on seeds heat treated at different temperatures: A) 250, B) 300, C) 350, D) 400, and E) 450°C. The samples were hydrothermally grown for 4 h at 80°C.

**Figure 7:**
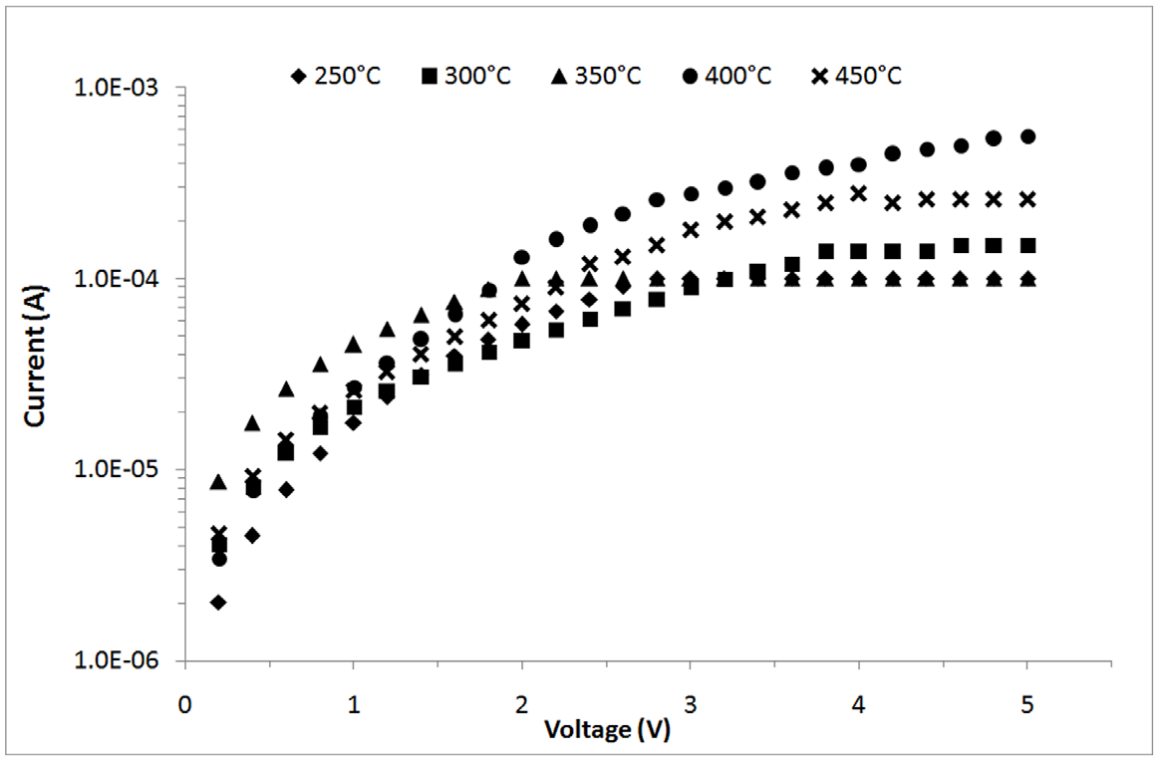
I–V curves of ZnO nanorods grown on seeds heat treated at different temperatures under UV illumination (λ = 375 nm).

Photoluminescence (PL) spectra of the ZnO nanorods were obtained at room temperature using a Jobin Yvon Horiba HR800UV System with a 325 nm line HeCd laser at a power of 20 mW. PL measurement enables the investigation of the electronic structure and optical characteristics of semiconductor materials by generating information on surface oxygen vacancies, defects, as well as separation and recombination of photoinduced charge carriers. The PL spectra of ZnO are normally composed of two parts. The first part is related to emission induced by the excitonic recombination of energy state around the bandgap related to the free and bound excitons [21]. The second part represents the defect-related band within the visible range. The main candidates are native defects such as oxygen vacancies (V_o_), zinc vacancies (V_Zn_), oxygen interstitial (O_i_), and oxygen antisite (O_Zn_) [21]. Figure 5 shows the PL spectra of ZnO nanorods grown on seeded substrates heat treated at different temperatures. Three luminescence emission peaks are observed, namely, a sharp UV emission at ∼380 nm, a green emission at ∼557 nm, and a yellow emission at ∼583 nm. The peak intensity of UV emission initially increases with increased heat-treatment temperature of seed, and then reaches a maximum at 400°C. Further increased temperature to 450°C results in rapidly decreased intensity. This finding shows that the near-band-edge emission intensity of the ZnO nanorods is influenced by the heat-treatment temperature of the seeds, which is closely associated with the crystal quality of the ZnO nanorod samples. This result is comparable with the XRD results in Figure 4, which shows increased (002) peak intensity with increased heat-treatment temperature of ZnO seeds. However, for the sample grown on seeds heat treated at 450°C, the PL spectrum is not comparable with the corresponding XRD pattern (Figure 4). The high (002) peak intensity for ZnO nanorods can be due to ZnO nanorods growth along the c-axis rather than to improved ZnO crystallinity. Figure 5 shows a strong, deep-level emission related to a yellow-green emission band with two peaks at 560 and 580 nm. Deep-level emission usually results from the radiative recombination of a photogenerated hole with an electron occupying an oxygen vacancy [22]. The deep-level emission intensity increases with increased heat-treatment temperature of the seeded substrate from 250°C to 350°C, decreases at 400°C, and then increases back to 450°C. A higher green PL intensity is due to the presence of a higher number of ionized oxygen vacancy defects [9]. An oxygen vacancy results from the competition between O atoms entering the ZnO lattice and those exiting this lattice by evaporation. At low-temperature heat treatment of seeds, the adsorption (in) rate of O atoms in the ZnO lattice is much faster than the evaporation (out) rate of ZnO from the lattice. O atoms have lower kinetic energy in the ZnO lattice at low temperature. Similar results were observed by Meng et al. [22]. With increased heat-treatment temperature of seeds, the kinetic energy of atoms in the ZnO lattice increases, which consequently increases the evaporation (out) rate of O atoms to exceed the adsorption rate and create more vacancies in the ZnO lattice [22]. However, for ZnO nanorods grown on seeds heat treated at 400°C, the intensity of deep-level emission is lower than 350°C. Theoretically, the intensity should be higher than 350°C. The observation indicates that the density of oxygen vacancies in the ZnO seed layer can be controlled to some extent by controlling the heat-treatment temperature of the ZnO seed layer. Khusaimi et al. [23] observed similar PL spectra that show a noticeable deep-level emission band for ZnO nanorods prepared by the hydrothermal method. For the as-grown ZnO nanorods prepared by the hydrothermal method, the chemical component of the ZnO nanorods is non-stoichiometric and usually comprises excess Zn atoms and oxygen vacancies [24].

**Figure 8:**
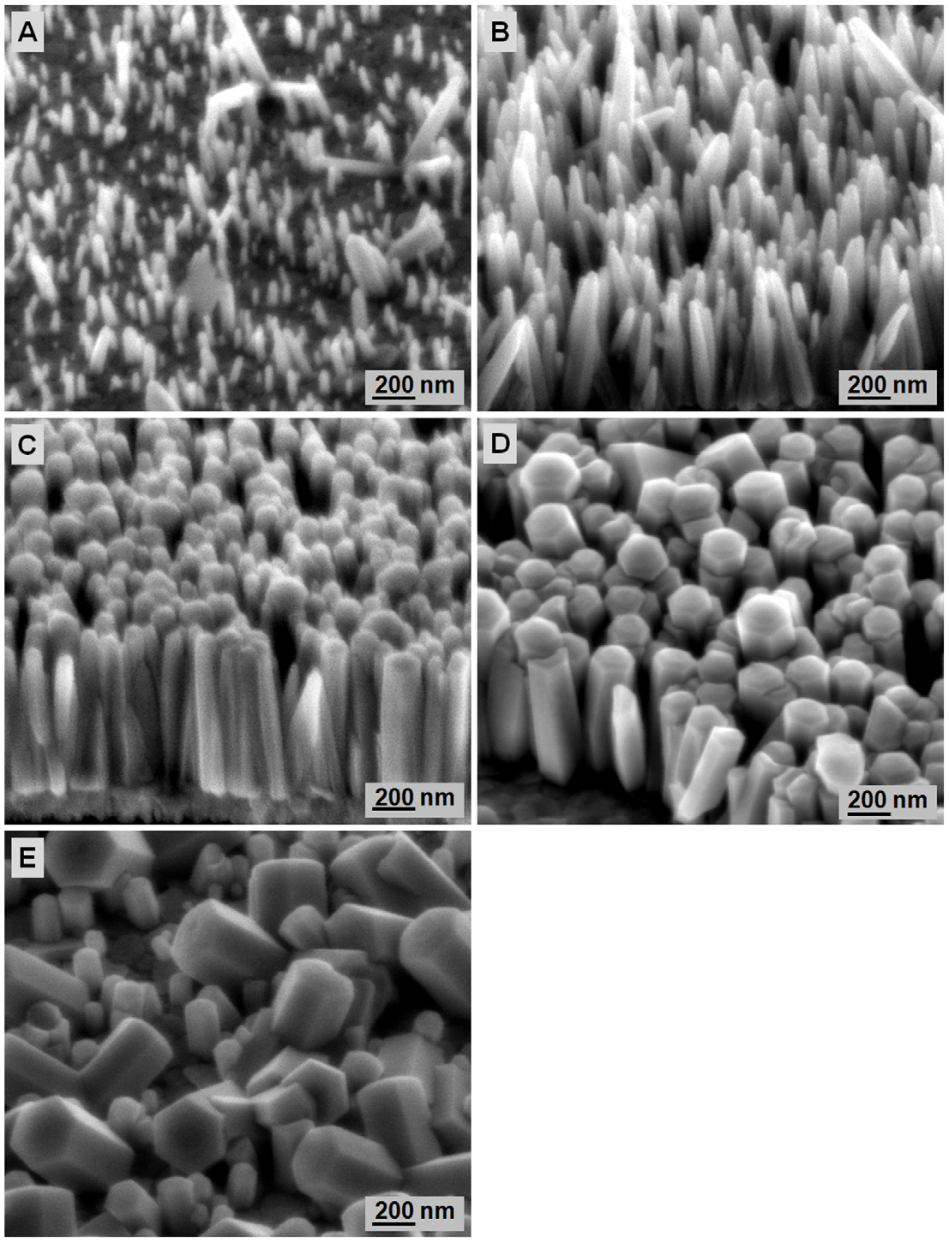
FESEM images of ZnO nanorods formed at different zinc nitrate concentrations: A) 0.05, B) 0.1, C) 0.2, D) 0.3, and E) 0.4 M. The samples were hydrothermally grown for 4 h at 80°C.

**Figure 9:**
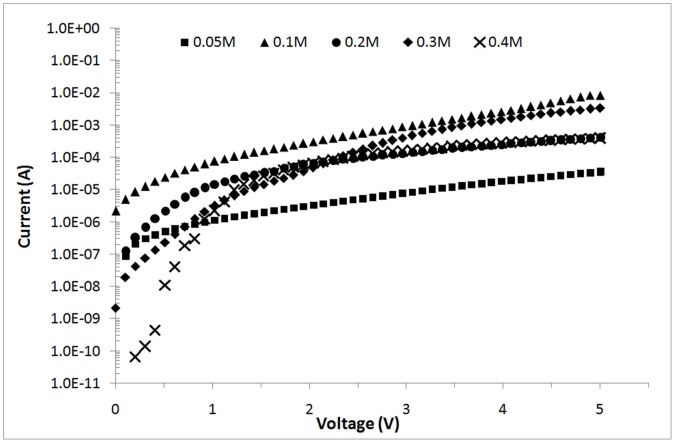
I–V curves of ZnO nanorods grown at different zinc nitrate concentrations under UV illumination (λ = 375 nm).

Figure 6 shows Raman spectra from 200 cm^−1^ to 700 cm^−1^ for ZnO nanorods grown at different temperatures. The peaks located at 303.3 and 520.5 cm^−1^ are attributed to the Si-substrate vibration modes [10]. Wurtzite ZnO crystal contains two formula units in the primitive cell that belong to the C_6_^4^ space group. According to the Brillouin zone, a single crystalline ZnO consists of eight sets of optical phonon modes with the existence of the following modes [2], [10]:
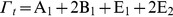
(1)


Non-polar phonon modes with E_2_ symmetry include two frequencies, namely, E_2_ (H) and E_2_ (L), which are associated with oxygen atoms and the Zn sublattice, respectively. The A_1_ and E_1_ modes are polar; they consist of transverse optical (TO) and longitudinal optical (LO) phonons. According to the selection rule, only E_2_ and LO are Raman active, whereas E_1_ and TO are forbidden. The E_2_ (H) mode is observed at 437.6 cm^−1^ (Figure 6), which is an intrinsic characteristic of the Raman-active mode of wurtzite hexagonal ZnO. The Raman spectrum measurement result is comparable with that of the XRD analysis, which shows a single peak at (002) corresponding to the wurtzite structure of ZnO. The Raman spectra obtained in this study agree with those of Li et al. [10]. However, our Raman and PL spectra are inconsistent with each other. The peak of E_1_ (LO) at 580 cm^−1^ is not detected in all samples, which can be attributed to defects such as O vacancies, Zn interstitials, or their complex. These defects are related to the high intensity and broad deep-level band observed in the PL spectra (Figure 5) [10]. This finding is due to the lattice mismatch between ZnO and substrates, which results in the stress effect. The occurrence of stress can cause phonon shifting and confer difficulty in analyzing Raman scattering properties [15].

**Figure 10:**
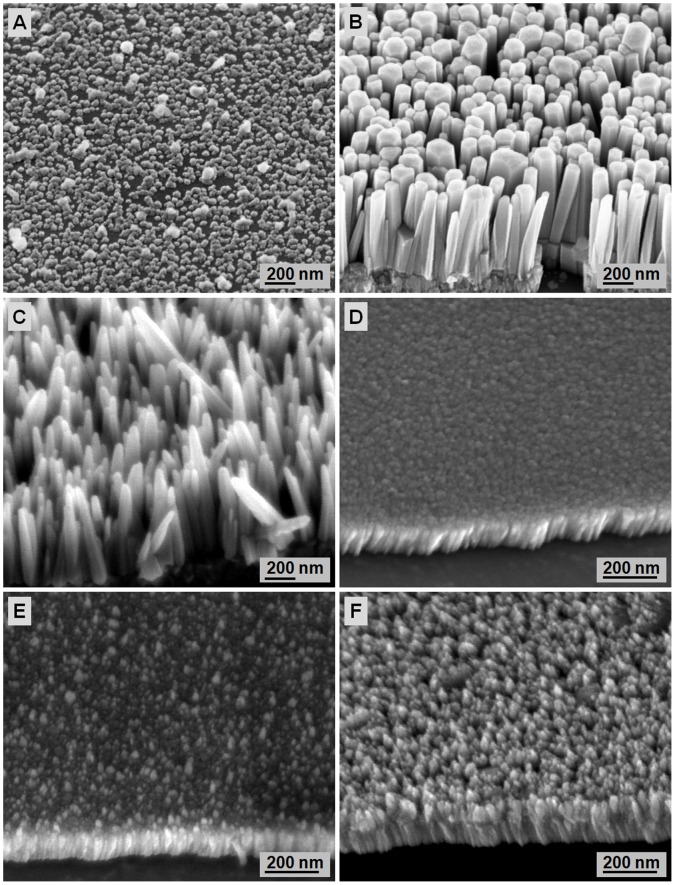
FESEM images of ZnO formed in a hydrothermal solution containing 0.1 M Zn(NO3)2 and 0.1 M HMT at different pΗ values: A) 3, B) 5, C) 7, D) 9, E) 11, and F) 13. The samples were hydrothermally grown for 4 h at 80°C.

**Figure 11:**
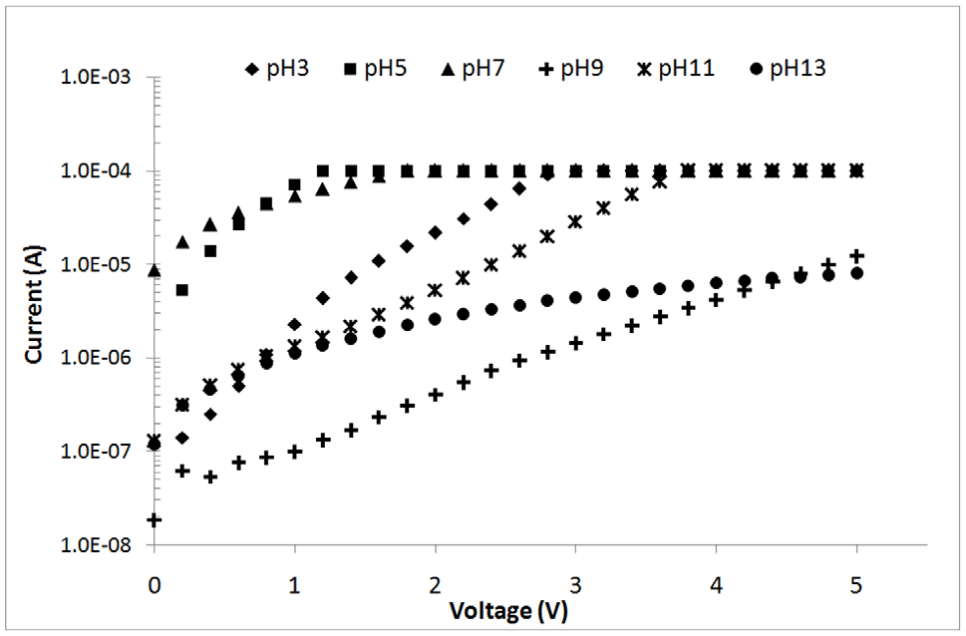
I–V curves for ZnO nanorod sensors under UV illumination (λ = 375 nm) at different hydrothermal solution pΗ values.

Figure 7 shows the *I*–*V* curves for the UV sensing of the fabricated ZnO nanorods grown on seeds heat treated at different temperatures measured under UV illumination (*λ* = 375 nm). With a 3 V applied bias, the photocurrent values measured are 1.0×10^−4^, 8.94×10^−5^, 1.01×10^−4^, 2.8×10^−4^, and 1.8×10^−4^ A at the seed-heat-treatment temperatures of 250, 300, 350, 400, and 450°C, respectively. The photocurrent values for ZnO nanorods grown on the seeded substrates heat treated at 400°C are the highest among all samples because of the larger surface area that eases oxygen adsorption and desorption on the surface [13], [18]–[19]. The surface area is determined from the average aspect ratio (average length/average diameter). The aspect ratios of the ZnO nanorods are 1.45, 2.37, 2.50, 9.16, and 7.73 for the samples heat treated at 250, 300, 350, 400, and 450°C, respectively. The sample heat treated at 400°C had the highest aspect ratio that contributed to the highest photocurrent values. Apart from the photoexcited electron-hole pair mechanism, oxygen adsorption and desorption on the surface is important for increasing or decreasing the conductivity of ZnO nanorods [19]. In darkness (without UV), oxygen is adsorbed onto the nanorod surface and negatively charged by capturing free electrons from ZnO, thereby leaving depleted region near the surface [20]. The negative oxygen ions are not free carriers and do not contribute to the nanorods conductivity. Negative oxygen ions can also introduce a depletion zone that can cause poor carrier mobility. These negative oxygen ions adhere onto the nanorods surface.

**Figure 12:**
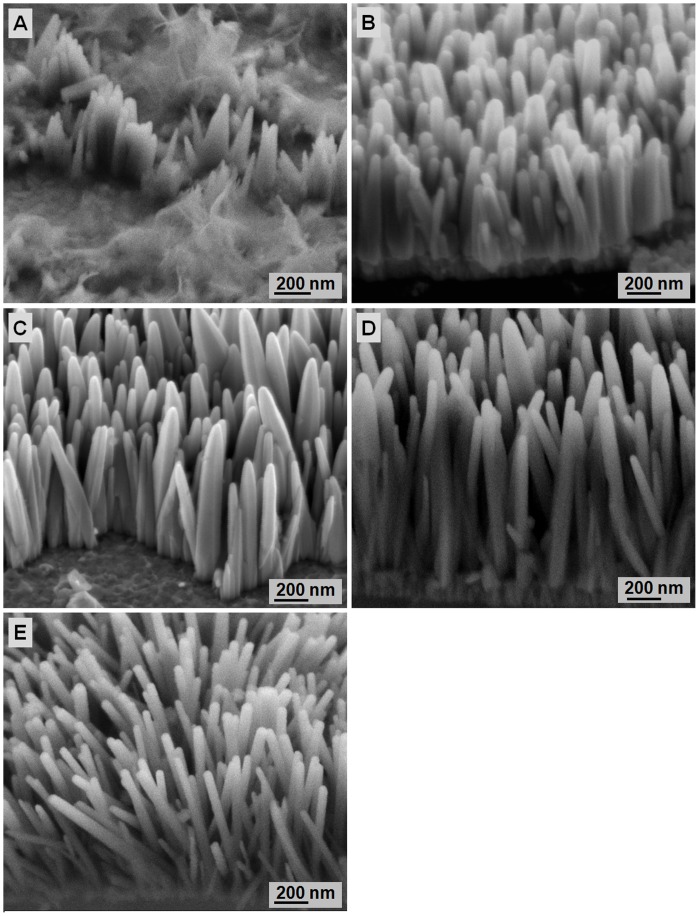
FESEM images of ZnO nanorods formed at 80°C for different hydrothermal growth durations: A) 1, B) 4, C) 8, D) 16, and E) 24 h.

**Figure 13:**
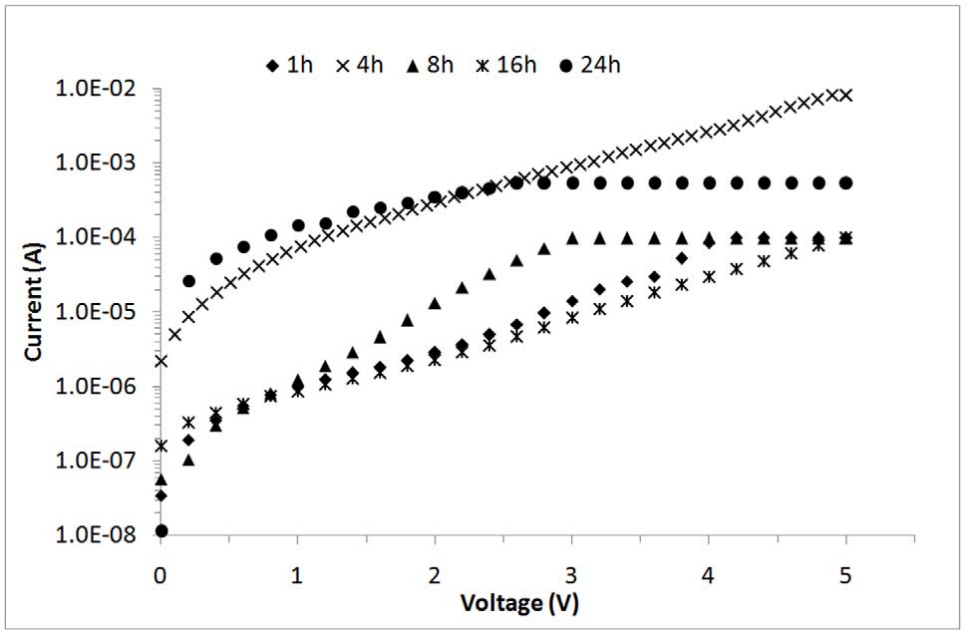
I–V curves for ZnO nanorod sensors under UV illumination (λ = 375 nm) at different hydrothermal growth durations.

When the ZnO nanorods are exposed to UV light, electron-hole pairs are generated. This phenomenon occurs when the photogenerated holes are captured by the negative oxygen ions through surface electron-hole recombination. Increased number of photogenerated electrons contributes to increased sensor conductivity. Oxygen photodesorption also lowers the barrier heights of grain boundaries and increases carrier mobility [18]–[19], [21]. Therefore, high surface-to-volume ratios of nanorods affect their photocurrent and UV-sensing properties.

**Figure 14:**
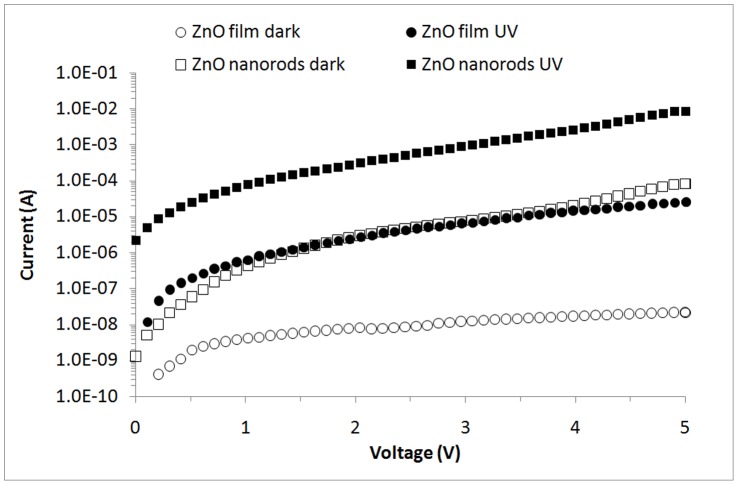
I–V curves for the ZnO film and ZnO nanorod sensors under UV illumination (λ = 375 nm).

### 3.2 Effect of the Zn(NO_3_)_2_ Concentration on ZnO Nanorod Growth

The effect of the Zn(NO_3_)_2_ concentration on the final morphology of ZnO nanorods formed is studied by varying the Zn(NO_3_)_2_ concentration: 0.05, 0.1, 0.2, 0.3, and 0.4 M. The heat-treatment temperature used to produce the ZnO seed layer is fixed at 400°C. The average diameter and length of the ZnO nanorods produced in 0.05 M Zn(NO_3_)_2_ are 79.6 and 320.7 nm, respectively. At low Zn(NO_3_)_2_ concentrations, the ZnO rods are inhomogeneous (Figure 8A) because of the low concentration of Zn^2+^ ions that result in less formation of Zn(OH)_2_, which further transforms to ZnO upon dehydration. At low concentrations, the supersaturation level is low. Thus, the nucleation and growth rates are relatively slow, which restrains rods growth at the nuclei sites. This result agrees with that of Khusaimi et al. [23]. Further increased Zn(NO_3_)_2_ concentration to 0.1 M results in more homogeneous ZnO nanorods with an average diameter of 81 nm and length of 770 nm (Figure 8B). Zn(NO_3_)_2_ and HMT (0.1 M each) in the hydrothermal bath produces similar concentrations of Zn^2+^ and OH^-^ ions in the bath. Thus, the ZnO nucleation rate increases and leads to mass rods growth. Song et al. [11] similarly observed that the aspect ratio of grown ZnO nanorods is determined by the relative growth rate of the polar surface and non-polar surface. Increased Zn^2+^ ions increase the amount of Zn(OH)_2_ produced, which increases the ZnO nanorods growth rate [10]. These endothermic growth processes hinder ZnO nanorods growth in the c-axis direction, thereby producing thicker and shorter nanorods. Further increased Zn(NO_3_)_2_ concentrations to 0.2, 0.3, and 0.4 M decrease the average rods length but increase the average rods diameter, as shown in Figures 8B to 8D, respectively.

High surface-to-volume ratios enable ZnO nanorods to be effectively used in UV sensing because they enhance the photo-response sensitivity. The effect of the high surface-to-volume ratio can be correlated with the final morphology of ZnO nanorods formed by hydrothermal reaction. The final morphology of these nanorods is known to be influenced by the hydrothermal conditions. Accordingly, the *I*–*V* behavior of ZnO nanorods for UV sensing formed at different Zn(NO_3_)_2_ concentrations is characterized by electrical measurements under UV light. Figure 9 shows that all samples respond to UV light but with different sensing characteristics. With a 3 V applied bias, the photocurrents obtained are 9.31×10^−6^, 1.08×10^−3^, 1.57×10^−4^, 5.61×10^−4^, and 1.77×10^−4^ A for 0.05, 0.1, 0.2, 0.3, and 0.4 M Zn(NO_3_)_2_, respectively. The highest photocurrent value is observed for ZnO nanorods grown in 0.1 M Zn(NO_3_)_2_ at 3 V. This finding is due to the higher specific area of these nanorods than the others. Figure 8B shows that the nanorods formed at this concentration are thinner and relatively longer, resulting in a high surface-to-volume ratio of nanorods than those formed at other concentrations. The responsivity, R was calculated by:
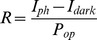
(2)Where I_ph_ is the photocurrent, I_dark_ is the dark current, and P_op_ is the optical power of the UV light [19]. The responsivity *R* of the fabricated UV-sensing devices at 3 V are 2.35×10^−5^, 0.026, 3.92×10^−3^, 3.12×10^−3^, and 4.42×10^−3^ A/W for 0.05, 0.1, 0.2, 0.3, and 0.4 M Zn(NO_3_)_2_, respectively. The high responsivity of ZnO nanorods produced with 0.1 M Zn(NO_3_)_2_ confirms the influence of the final morphology of ZnO nanorods on the oxygen chemisorption because more surfaces are available for oxygen adsorption/desorption upon air exposure.

### 3.3 Effect of the Hydrothermal Bath Solution pΗ

For ZnO nanorods growth, the hydrothermal reaction pΗ is crucial because OH^−^ is strongly related to the reactions that produce ZnO structures. The effects of pΗ on the microstructure and properties of ZnO nanorods are determined by varying the pH (3, 5, 7, 9, 11, and 13). The heat-treatment temperature and Zn(NO_3_)_2_ concentration are fixed at 400°C and 0.1 M, respectively. The corresponding FESEM images of the grown ZnO at each pΗ are shown in Figure 10. The ZnO nanorods only form at pΗ 5 and 7. ZnO nanorods are known to erode in acidic condition, and the final growth depends on the competition between growth and etching [25]. In this work, HCl is used to adjust the pH of the hydrothermal bath solution. The Cl^−^ ions can etch the ZnO seed layer and decrease the nucleation sites. Thus, heterogeneous nucleation cannot occur and ZnO nanorods formation is impeded. On the other hand, the H^+^ ions increase the competition between Zn^2+^ and H^+^ to react with OH^−^. OH^−^ ions are more preferred to react with H^+^ to produce H_2_O compared with Zn^2+^. Thus, less Zn(OH)_2_ is formed, leading to decreased ZnO nanorods formation. Hexagonal flat-top nanorods with well-defined angular walls are observed in Figure 10B. This finding is due to the slow growth rate of ZnO crystals. At pΗ 5, Zn^2+^ ions compete with H^+^ ions to react with OH^−^. As a result, Zn and O atoms move to the right of the crystal and create hexagonal rods. Circular rods are formed without pΗ adjustment (Figure 10C). The rod shape of ZnO is generally assumed to result from a dissolution-recrystallization mechanism or nanoparticle-oriented aggregation [26]. For a Zn(NO_3_)_2_/HMT system, precipitation more rapidly occurs and produces spherical ZnO. The spherical particle size is critical because the particles fuse to form embryonic rod-like microcrystals. The particles diffuse at the c-axis because of the net polarization. Rapid dissolution-recrystallization causes ZnO to form spherical particles. Thus, rod-shaped ZnO is formed. Figures 10D to 10F show no ZnO nanorods formation at pΗ 9, 11, and 13, respectively. Theoretically, Zn(OH)_2_ formation increases with increased OH^−^ concentration, further induces the growth of ZnO nanorods. However, in this work, no ZnO nanorods formation is observed because of the competition between ZnO growth and erosion during hydrothermal reaction. Wurtzite ZnO crystal is typically a polar crystal at the axial direction, which is unstable compared with the side planes that are non-polar and more stable. When the OH^−^ concentration in solution exceeds the critical concentration, the ZnO growth rate decreases and the dissolution of ZnO nanorods increases. Given that the polar plane (top of rod) is unstable, erosion occurs on the polar plane and nanorods walls. Consequently, the ZnO nanorods appear to merge and form ZnO films. Higher OH^−^ concentrations accelerate the dissolution of ZnO, as shown in Equation 3 [10]:
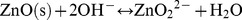
(3)


In the presence of low OH^−^, ZnO nanorods are not formed because of insufficient Zn(OH)_2_ formation. However, further increased OH^−^ concentration decreases the growth rate because of the simultaneous dissolution of ZnO nanorods in the presence of excess OH^−^. Therefore, a threshold pΗ level must exist to form ZnO nanorods.

Apart from having a high surface-to-volume ratio, the ZnO nanorods formed in this work also show good crystallinity at the (002) peak. This property is correlated with the preferred orientation at the c-axis, which is influenced by the hydrothermal solution pH. According to Mamat et al. [17], highly crystalline nanorods have enhanced photoresponse values. Figure 11 shows that a forward bias of 3 V at pΗ 5 and 7 results in a higher photocurrent than those formed at other pΗ values. This finding can be due to the nanorods morphology (Figure 11) and high crystallinity (data not shown), in agreement with the report of Gosh and Basak [16]. Orderly oriented crystallites increase the surface state of nanorods because more free electrons can be trapped. Surface states acting as trap states capture free electrons, which leads to surface-electron depletion with a band bending upwards toward the surface. A high crystallinity facilitates easy electron transport through the nanorods. Electron transport is influenced by several factors, including crystal quality and crystallographic orientation [16]. With the good crystallographic orientation and high crystallinity of the ZnO nanorods formed at pΗ 5 and 7, the photogenerated electron can be directly transported by a diffusive transport mechanism. This mechanism significantly reduces the recombination losses at photogenerated charge carriers through the nanorods, and further increases the responsitivity of UV sensing [16]. The responsivities at 3 V are 3.3×10^−5^, 1.12×10^−3^, 2.5×10^−3^, 3.61×10^−5^, 7.11×10^−5^, and 1.13×10^−5^ A/W for pH 3, 5, 7, 9, 11, and 13, respectively. The high responsivity values at pH 7 prove that the high crystallinity of ZnO nanorods positively affects the UV-sensing responsivity.

### 3.4 Effect of the Hydrothermal Growth Duration on ZnO Nanorod Growth

Figure 12 shows the FESEM images of ZnO nanorods grown for different hydrothermal durations. Figure 12A shows that at a growth duration of 1 h, ZnO nanorods formation is less and inhomogeneous. The diameter of nanorods is <45 nm, with an average length shorter than 500 nm. A 1 h growth duration is insufficient for the complete thermal decomposition of HMT and Zn(NO_3_)_2_ to provide OH^−^ and Zn^2+^ ions, respectively, and enable ZnO formation. Thus, fewer nanorods are formed because of insufficient starting materials. A small amount of Zn^2+^ ions leads to a slower nucleation rate, which induces smaller rods growth [22]. Further prolonged growth duration results in denser and more homogeneous nanorods with average lengths of 770 nm, 865 nm, 948 nm, and 1.1 µm for growth durations of 4, 8, 16, and 24 h, respectively. The continuation of the thermal decomposition of HMT and Zn(NO_3_)_2_ with prolonged duration increases the number of OH^−^ and Zn^2+^ that are attracted toward the polar surface of seeds and form ZnO in the c-axis direction. Increased length with increased growth duration is due to the crystal habits of wurtzite ZnO itself. Wurtzite ZnO rapidly grows following the [0001] plane. However, with prolonged growth duration, the concentrations of the zinc precursor and HMT are depleted because most chemicals are consumed. Consequently, the dissolution reaction proceeds faster and competes with the crystallization process until a state of equilibrium is reached. High dissolution occurs in the [0110] plane (side of rods) because of the high surface area compared with the [0001] plane. Given that the ZnO growth rate is preferentially high at the c-axis, ZnO recrystallization causes growth on the [0001] plane. Thus, thinner and longer ZnO nanorods are produced with increased growth duration [5].

The hydrothermal growth duration is believed to affect the final morphology of ZnO nanorods. Figure 13 shows an *I*–*V* curve of the fabricated ZnO nanorods growth at different hydrothermal reaction durations. The calculated responsivity are 3.54×10^−4^, 0.024, 1.47×10^−3^, 1.84×10^−4^, and 0.013 A/W for hydrothermal growth durations of 1, 4, 8, 16, and 24 h, respectively. ZnO nanorods grown for 4 h have the highest responsivity among all other samples. Theoretically, ZnO nanorods grown for 8, 16, and 24 h should have the best responsivity because of their high surface areas. However, the crystallinity of ZnO nanorods grown for 4 h is the highest possibly because of the responsivity of the sample. Highly crystalline ZnO nanorods cause less carrier scattering because the photogenerated electrons can be directly transported.

Figure 14 shows the *I*–*V* curves of the fabricated device using conventional ZnO film and ZnO nanorods (synthesis at optimum parameters). With a 3 V applied bias, the photocurrent measured for ZnO film and ZnO nanorods are 6.9×10^−6^ and 9.7×10^−4^ A, respectively which is high by two orders of magnitude. This result is due to the large surface-area-to-volume of the nanorods compared with the film [19], [17]. The higher surface area of nanorod structures than the thin film is important in UV sensing because it involves the surface reactions between free carriers and the surrounding environment, such as oxygen. Similarly, Ji et al. [18] found that the photocurrent generated from ZnO nanorods is higher than conventional ZnO thin film. However, in this study, the photocurrent is 8.23×10^−3^ A at a 5 V bias, which is higher than the 2.99×10^−3^ A found by Ji et al. [18].

As aforementioned, the surface area plays an important role in the UV-sensing mechanism. The sensing mechanism involves a surface reaction between free carriers and the surrounding environment, including oxygen and humidity, which are mostly governed by oxygen adsorption and desorption during UV illumination [17]–[20]. In darkness, oxygen molecules from the surrounding environment are easily adsorbed onto ZnO surface by capturing free electrons from ZnO as in reaction (4), where O_2_ is an oxygen molecule, e^−^ is a free electron, and O_2_^−^ is adsorbed oxygen onto the nanorods surfaces [17]. When the ZnO nanorods are exposed to UV light, electron-hole pairs are generated as in reaction (5), where *hv* is the photon energy, e^−^ is the photogenerated electron, and h^+^ is the photogenerated hole. Subsequently, photogenerated holes recombine with adsorbed oxygen ions and produce oxygen molecules, which then desorb from the nanorods surfaces as in reaction (6). Simultaneously, the increase in photogenerated electrons causes an accumulation of conduction electrons, and the produced oxygen lowers the depletion layer. As a result, carrier mobility increases. With an applied bias voltage, the free carriers move toward electrodes that generate the photocurrent. The higher surface area of ZnO nanorods than ZnO film causes high rates of oxygen molecule absorption and diffusion onto the nanorods surface upon UV light exposure [17].
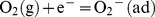
(4)

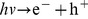
(5)

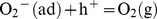
(6)


### Conclusions

In this work, the effects of synthesis parameters on the structure and morphology of ZnO nanorods grown on a heat-treated seeded template were studied. The optimum parameters for ZnO nanorods growth using hydrothermal reaction were obtained on a seed layer under the following conditions: heat treatment at 400°C for 10 min, 0.1 M Zn(NO_3_)_2_, pΗ 7 of hydrothermal bath solution, and 4 h of growth duration. High surface-to-volume ratios of ZnO nanorods produced excellent UV-sensing responsivities. The highest responsivity of 0.024 A/W was obtained for ZnO nanorods synthesized using the optimum synthesis parameters.
